# Consensus statement on the management of Achondroplasia in the Middle East

**DOI:** 10.3389/fendo.2026.1888346

**Published:** 2026-09-17

**Authors:** Nandu Thalange, Tawfeg Ben Omran, Afaf Alsagheir, Khalid Hussain, Maha Faden, Mona Aglan, Sareea Alremeithi, Abdelhadi Habeb

**Affiliations:** 1 Pediatric Endocrinologist, Genesis Healthcare, Dubai, United Arab Emirates; 2 Medical Geneticist, Sidra Medicine, Doha, Qatar; 3 Pediatric Endocrinologist, King Faisal Specialist Hospital & Research Centre, Riyadh, Saudi Arabia; 4 Pediatric Endocrinologist, Sidra Medicine, Doha, Qatar; 5 Medical Geneticist and Metabolic Specialist, King Saud Medical City, Riyadh, Saudi Arabia; 6 Professor of Clinical Genetics, National Research Centre, Cairo, Egypt; 7 Pediatric Endocrinologist, Sheikh Khalifa Medical City, Abu Dhabi, United Arab Emirates; 8 Pediatric Endocrinologist, National Guard Hospital, Madinah, Saudi Arabia

**Keywords:** FGFR3 testing, medical genetics, middle east guidelines, pediatric endocrinology, skeletal dysplasia, vosoritide

## Abstract

Achondroplasia is the most common skeletal dysplasia and presents as a lifelong, multi-system condition characterized by disproportionate short stature, rhizomelic limb shortening, macrocephaly with frontal bossing, and a spectrum of neurological, respiratory, orthopedic, and psychosocial complications. Effective management requires anticipatory surveillance, coordinated multidisciplinary care, and early recognition of life-threatening complications. Therapeutic options have expanded in recent years with the introduction of vosoritide, a C-type natriuretic peptide analog that targets the underlying pathophysiology and has demonstrated significant improvements in linear growth and body proportionality in eligible children. However, global advances have not been uniformly translated into clinical practice across the Middle East, where challenges such as delayed diagnosis, inconsistent access to genetic testing, limited multidisciplinary infrastructure, and inequities in treatment availability continue to impact patient outcomes. In this document, a panel of pediatric endocrinologists and clinical geneticists from the Middle East convened to develop a set of regionally relevant, evidence-informed consensus statements on the diagnosis and management of achondroplasia in light of novel advances in the management of this condition. Using a modified Delphi process, the panel achieved consensus on 25 statements covering diagnosis, surveillance, multidisciplinary management, psychosocial support, pharmacologic interventions, transition to adult care, and research priorities. This consensus, endorsed by the Arab Society for Pediatric Endocrinology and Diabetes (ASPED) and the MENA Medical Genetics Association, aims to standardize and enhance the quality of care for individuals with achondroplasia across the region by integrating international best practices with local healthcare realities. The author panel believes that the implementation of these recommendations has the potential to improve early detection, optimize long-term outcomes, strengthen family support, and expand equitable access to emerging therapies.

## 1 Introduction

Achondroplasia is the most common form of skeletal dysplasia, with a worldwide prevalence of around 4.6 per 100,000 live births (1, 2). Its estimated prevalence is much higher in the Middle East and North Africa: 34.31 per 100,000 live births according to a meta-analysis published in 2020 (3). However, reliable population-based epidemiological data from the region remain scarce, and this value should be cautiously interpreted as an estimate based on available published studies rather than definitive registry data. Consequently, the reported prevalence should not be considered a definitive estimate for the Middle East and North Africa, and further registry-based and population-level studies are needed to better characterize the true epidemiology of achondroplasia in this population.

Achondroplasia is caused by a heterozygous gain-of-function variant in the fibroblast growth factor receptor 3 (*FGFR3*) gene, most commonly the p.Gly380Arg variant, resulting in overactive signaling and impaired cartilage cell proliferation and maturation (4). Its inheritance is autosomal dominant; however, most cases are caused by *de novo* mutations (5). Achondroplasia is characterized clinically by disproportionate short stature, rhizomelic limb shortening, macrocephaly with frontal bossing, and midface hypoplasia (6, 7). Beyond its skeletal features, achondroplasia is a multi-system condition associated with foramen magnum stenosis, obstructive sleep apnea, recurrent otitis media, spinal stenosis, obesity, and functional limitations across daily activities (8, 9). These complications evolve across the lifespan, requiring anticipatory guidance, coordinated multidisciplinary care, and early detection to prevent morbidity.

Management strategies for achondroplasia have historically focused on supportive and surgical interventions, including management of ear, nose, and throat (ENT) complications, neurosurgical decompression when indicated, orthopedic correction of lower limb deformities, nutritional guidance, and physiotherapy. Recently, therapeutic options have expanded with the approval of vosoritide, a C-type natriuretic peptide (CNP) analog that enhances endochondral bone growth by downregulating overactive FGFR3 signaling. Clinical trials and real-world data demonstrate that vosoritide increases annualized growth velocity (AGV) and improves body proportionality, with a favorable safety profile in eligible children (10–12). Despite these advances, management remains complex, and treatment decisions require shared decision-making informed by clinical evidence and healthcare system constraints.

In the Middle East region, several challenges hinder optimal care delivery in achondroplasia. Key unmet needs include delayed diagnosis, inconsistent access to genetic testing, lack of formal multidisciplinary teams, and differences in care pathways across countries. Disparities in healthcare access, particularly between citizens and expatriates, further impact the availability and reimbursement of high-cost therapies such as vosoritide. Moreover, regional epidemiological data and real-world evidence are limited, hindering the development of contextually relevant care models. The objective of this consensus is to provide evidence-based statements developed by pediatric endocrinologists and clinical geneticists from across the Middle East. These statements integrate international guidance with regional clinical experience to offer a practical and culturally relevant framework for improving the lifelong management of individuals with achondroplasia in the region.

## 2 Methods

### 2.1 Assembly of expert panel

An expert panel (comprising the author group) was established, including five consultant pediatric endocrinologists and three genetic/metabolic specialists with clinical experience in the management of achondroplasia from across the Middle East and Egypt (Qatar, UAE, KSA, and Egypt). The average number of years of clinical experience among members of the panel was 29. The panel convened on May 3, 2025 to discuss the landscape of achondroplasia management in the region in the context of screening and diagnosis, treatment options, current unmet needs, and opportunities to optimize care.

### 2.2 Literature review

A literature review was conducted between May 5, 2025 and June 6, 2025 using PubMed to identify pertinent treatment guidelines, randomized controlled trials, observational studies, and systematic reviews to allow for the generation of evidence-based consensus statements. The literature review included articles published between January 2015 and June 2025 using the following search string: ((“achondroplasia”[MeSH Terms] OR “skeletal dysplasia”[All Fields]) AND (“management”[All Fields] OR “treatment”[All Fields] OR “therapy”[Subheading] OR “guideline”[Publication Type] OR “practice guideline”[Publication Type] OR “observational study”[Publication Type] OR “randomized controlled trial”[Publication Type] OR “systematic review”[Publication Type] OR “observational”[All Fields])). Animal studies, publications before 2015, and studies in any language besides English were not considered.

This targeted literature review was undertaken to inform the development of the consensus statements rather than to perform a formal systematic review or evidence synthesis. In addition to the PubMed search, the author panel considered key publications identified through reference lists of relevant articles, international consensus documents and clinical practice guidelines, and regulatory prescribing information for vosoritide (including FDA, EMA, and regional labels where available) to ensure that the recommendations reflected the most current evidence and regulatory landscape.

### 2.3 Development of consensus statements

Consensus statements relating to the management of achondroplasia were initially developed by the first author based on findings from the literature review, key recommendations from international guidelines, and areas of unmet need identified during the expert panel meeting on May 3, 2025. Draft statements were circulated to all authors for review and refinement prior to formal voting.

The Delphi technique was used to reach consensus on all statements, with respondents anonymously voting through an online platform. The Delphi process was not designed with a predefined number of voting rounds; rather, additional rounds were to be undertaken until consensus was achieved for all statements. Voting options were:

- “Strongly agree”
- “Agree”
- “Neither agree nor disagree”
- “Disagree”
- “Strongly disagree”
- “Abstain/unable to answer”

Participants were also invited to provide qualitative free-text comments and reasons for disagreement or suggested modifications for any statement. A consensus was considered achieved when >80% of experts selected either “strongly agree” or “agree”. The first author coordinated the Delphi process and served as the facilitator responsible for collating voting results and anonymized comments but did not participate in voting.

Statements that did not achieve consensus during the first voting round, or for which substantive feedback was provided, were reviewed by the author group. Suggested modifications were considered and incorporated where appropriate. A virtual consensus meeting was subsequently held on February 15, 2026 to discuss the voting outcomes, review areas of disagreement, and finalize revisions to the statements before a second round of anonymous voting. The revised statements were then redistributed through the online platform for re-evaluation.

## 3 Results

A total of 25 consensus statements were developed and disseminated to the expert voting group. These statements addressed diagnosis and early detection, goals of management, multidisciplinary care, anthropometry, pharmacological treatment, long-term management, and real-world data generation in achondroplasia.

Seven panel members participated in the Delphi process and completed the first round of voting. The first author served as the Delphi facilitator and coordinated statement development, voting administration, and collation of anonymized feedback, but did not participate in voting. Feedback provided through the online platform was reviewed, including comments relating to statement wording, implementation considerations, and clarification of recommendations. Eight statements did not achieve the predefined consensus threshold and/or received substantive feedback; these were revised following review by the author group and discussion during a virtual consensus meeting held on February 15, 2026.

A second and final round of anonymous voting was subsequently conducted using the revised statements. Consensus was achieved for all 25 statements within two rounds of voting, with agreement levels ranging from 86% to 100% (**Tables 1**–**6**).

**Table 1:** Consensus statements – diagnosis and early detection.

Consensus statement	Agreement level (%)
1.1 Achondroplasia should be suspected based on characteristic clinical features at birth, radiological findings, and confirmed with targeted *FGFR3* genetic testing.	100
1.2 Where available and clinically feasible, MRI of the craniocervical junction should be offered routinely in early infancy to screen for foramen magnum stenosis, with imaging reports incorporating standardized radiological scoring systems.	100
1.3 Where available and clinically feasible, polysomnography should be performed routinely to detect sleep-disordered breathing, including central sleep apnea, which may not be reliably identified through clinical assessment alone.	86
1.4 Prenatal diagnosis through ultrasound and molecular testing should be offered when there is clinical suspicion or a positive family history.	86
1.5 Prenatal diagnosis of achondroplasia should involve clear, non-directive counseling and the provision of balanced, evidence-based information to families, including education on positioning and handling of infants with achondroplasia.	100
1.6 Parents who receive a prenatal diagnosis of achondroplasia should be offered early psychological support and education.	100

**Table 2:** Consensus statements – goals of management and multidisciplinary care.

Consensus statement	Agreement level (%)
2.1 The primary goals of achondroplasia management are to anticipate, identify, and treat complications while supporting a healthy lifestyle, autonomy, self-esteem, and mental well-being.	100
2.2 Management of achondroplasia should involve a multidisciplinary team, ideally including a pediatric endocrinologist, geneticist, orthopedic surgeon, pulmonologist (for sleep apnea studies), neurologist/neurosurgeon, physiotherapist, dentist/orthodontist, audiologist, psychologist, dietitian, and occupational therapist.	100
2.3 A multidisciplinary care coordinator or lead clinician should be designated to ensure continuous care, effective communication, and follow up across specialties.	100
2.4 Surgical interventions should only be performed by clinicians with expertise in achondroplasia, and decisions should be made in conjunction with the multidisciplinary team.	86

**Table 3:** Consensus statements – monitoring and anthropometry.

Consensus statement	Agreement level (%)
3.1 Growth in infants and children with achondroplasia should be monitored longitudinally every 3–6 months using achondroplasia-specific growth charts for height, weight, and head circumference.	100
3.2 Anthropometric measurements should be performed consistently by the same trained clinician or nurse using standardized methods.	100
3.3 Regular screening and surveillance should be conducted for comorbidities including foramen magnum stenosis, sleep apnea, ENT problems, neurological issues, orthopedic complications, and obesity.	100

**Table 4:** Consensus statements – pharmacological treatment.

Consensus statement	Agreement level (%)
4.1 Vosoritide should be considered in eligible children with achondroplasia starting from birth or the age of 4 months, in alignment with regional and global regulatory approvals and clinical guidelines.	100
4.2 Initiation of vosoritide treatment should follow a structured and safety focused approach. Caregivers should receive comprehensive injection training from a specialist nurse, including instruction on injection technique and site rotation. The first dose should be administered under medical supervision with an observation period of approximately 1 hour to monitor potential adverse reactions. Patients and caregivers should also be advised to ensure adequate hydration prior to injection to reduce the risk of transient hypotension.	100
4.3 Clinicians should advocate for equitable access to vosoritide, including reimbursement mechanisms for both citizens and expatriates.	100
4.4 Treatment response to vosoritide should be monitored through standardized anthropometric measurements and clinical assessment at regular intervals, with calculation of annualized growth velocity. Where feasible, follow-up should be age-stratified, with assessments every 3 months in children aged 0–2 years, every 4–6 months in children aged 3–5 years, and every 6 months in children older than 5 years, to ensure timely evaluation of growth response, safety, and adherence.	100
4.5 Cessation of vosoritide treatment should be considered when annualized growth velocity falls below 1.5 cm/year despite adherence, or when epiphyseal closure is confirmed radiographically. Following treatment discontinuation, individuals should continue to receive ongoing surveillance for spinal and other achondroplasia-related complications and be supported through a structured transition plan to adult services, where appropriate.	100
4.6 Pharmacologic treatment decisions should involve shared decision making with families and incorporate cultural, financial, and healthcare system considerations.	100

**Table 5:** Consensus statements – long-term management and transition to adult care.

Consensus statement	Agreement level (%)
5.1 Follow-up of individuals with achondroplasia should adhere to specific guidelines that include risk-based monitoring for complications across all organ systems.	100
5.2 The use of quality of life tools is recommended to monitor patient wellbeing over time and guide individualized interventions.	100
5.3 Support groups should be promoted and empowered as part of long-term care, including the development of family-led peer support networks to facilitate information sharing, emotional support, and social connection.	100
5.4 Transition from pediatric to adult care should be planned in a structured manner, ideally through transition clinics involving both pediatric and adult specialists. This phase should also include guidance on employment and independent living.	100

**Table 6:** Consensus statements – real-world data and registries.

Consensus statement	Agreement level (%)
6.1 Establishing a regional data registry or participating in international registries is recommended to facilitate standardized data collection and global benchmarking.	100
6.2 Retrospective observational studies using pre-existing clinical data are encouraged as a practical and scalable approach to generate regional real world evidence.	100

## 4 Discussion

### 4.1 Diagnosis and early detection

#### 4.1.1 Initial suspicion

Achondroplasia, the most common form of disproportionate short stature, can often be recognized at birth, and in many cases, prenatally, owing to its distinctive clinical and radiographic features. Affected newborns typically present with rhizomelic limb shortening, macrocephaly with frontal bossing, midface hypoplasia, and redundant skin folds over the proximal limbs. Radiographically, characteristic findings include narrowing of interpedicular distances in the lumbar spine, shortened long bones with metaphyseal flaring, and a small foramen magnum, among others (1, 2, 7, 13).

Following clinical suspicion, molecular confirmation through targeted testing of the *FGFR3* gene is recommended. The c.1138G>A and c.1138G>C (p.Gly380Arg) variants account for over 95% of cases and can be reliably detected using focused assays, providing a sensitive and specific diagnostic approach (6, 14). In the Middle East, limited awareness of skeletal dysplasias, overlapping phenotypes, and constrained access to genetic testing in some areas often contribute to diagnostic delays. Promoting early clinical recognition and expanding access to confirmatory genetic testing are therefore crucial to enable early diagnosis and timely care planning.

In addition to genetic confirmation, early risk stratification for potentially life-threatening complications is critical in infancy. International guidelines recommend routine MRI of the craniocervical junction in early infancy, where available and feasible, to screen for foramen magnum stenosis, given the poor correlation between clinical signs and radiological severity (14–18). Use of standardized radiological scoring systems improves consistency of interpretation and supports timely neurosurgical referral when indicated. Additionally, polysomnography should be performed routinely in infancy, where practical, as central sleep apnea is common in achondroplasia and may be missed without formal sleep studies (5, 15, 18–21). Early identification of craniocervical compression and sleep-disordered breathing enables proactive intervention and can reduce morbidity and mortality in this population.

While routine MRI of the craniocervical junction and polysomnography represent the preferred standard of care, implementation may be influenced by local resource availability. In some settings across the Middle East, access to pediatric MRI services, anesthesia support, neuroradiology expertise, or dedicated sleep laboratories may be limited. In such circumstances, clinicians should maintain a low threshold for referral to specialist centers with appropriate expertise, particularly when clinical symptoms, neurological findings, growth patterns, or sleep-related concerns suggest an increased risk of complications. Efforts to strengthen referral pathways and access to specialized diagnostic services remain an important regional priority.

#### 4.1.2 Prenatal diagnosis

Prenatal diagnosis of achondroplasia should be considered when there is clinical suspicion on ultrasound or a positive family history. Typical ultrasonographic findings include rhizomelic limb shortening, macrocephaly with frontal bossing, and midface hypoplasia, which often become apparent after 24 weeks of gestation (1, 6, 18). When such features are detected, molecular confirmation provides rapid and definitive diagnosis (14, 15).

Early genetic confirmation enables appropriate perinatal planning, including delivery management and counseling on expected complications. Across global guidelines, prenatal testing is recommended to facilitate informed parental decision-making and preparedness. In the Middle East, improving access to genetic counseling and prenatal testing services remains a priority, requiring close collaboration between maternal-fetal medicine specialists, geneticists, and endocrinologists to ensure adequate implementation.

In addition, prenatal diagnosis of achondroplasia should always be accompanied by non-directive counseling that provides families with balanced, evidence-based information about the condition, expected outcomes, and available support resources. Counseling should emphasize that achondroplasia is compatible with a good quality of life (QoL) when managed through multidisciplinary care, while also addressing practical aspects such as safe positioning and handling of infants to prevent cervical compression and kyphosis (1, 8, 15).

Following a prenatal diagnosis, families should be offered early psychological support and tailored education to help them process the diagnosis, prepare for delivery, and establish realistic expectations (6, 14). International guidelines emphasize that counseling must remain empathetic, culturally sensitive, and non-directive. In the Middle East context, there is a need to strengthen psychosocial support systems and ensure access to trained genetic counselors who can provide reassurance and continuity of care support.

### 4.2 Goals of management and multidisciplinary care

#### 4.2.1 Goals of achondroplasia management

The management of achondroplasia extends beyond addressing growth and skeletal abnormalities, and should aim to anticipate, identify, and manage complications while promoting overall health and well-being. Common medical complications, such as foramen magnum stenosis, sleep apnea, spinal stenosis, otitis media, and obesity, require proactive surveillance and timely intervention through coordinated multidisciplinary care (1, 15, 18). Equally important are strategies that foster autonomy and psychosocial resilience, as individuals with achondroplasia may experience social stigma and reduced self-esteem (8, 22). In the Middle East, where psychosocial support services for rare conditions are still developing, integrating mental health professionals and patient advocacy networks into multidisciplinary teams can help achieve these holistic management goals.

#### 4.2.2 Multidisciplinary care

Optimal management of achondroplasia requires a multidisciplinary team that brings together diverse expertise to address the medical, developmental, and psychosocial aspects of care. Core members typically include a pediatric endocrinologist, clinical geneticist, orthopedic surgeon, pulmonologist, neurologist or neurosurgeon, physiotherapist, psychologist, dentist or orthodontist, audiologist, dietitian, and occupational therapist (1, 14, 15). Regular team reviews help ensure early detection and management of complications such as foramen magnum stenosis, sleep apnea, otitis media, spinal stenosis, and orthopedic deformities.

To maintain continuity and avoid fragmented care, appointing a designated care coordinator or lead clinician is recommended to oversee follow-up, referrals, and communication between specialties and families. Surgical interventions, including foramen magnum decompression, spinal decompression, or limb correction, should be performed only in centers experienced in achondroplasia, following multidisciplinary case discussions to optimize timing and reduce risk (8, 18). In the Middle East, multidisciplinary team structures are often informal or centralized in tertiary hospitals. Strengthening referral pathways, establishing regional multidisciplinary clinics, and creating clear care coordination frameworks are key priorities to improve long-term outcomes and patient care across the region.

### 4.3 Monitoring and anthropometry

#### 4.3.1 Anthropometric measurements

Longitudinal monitoring of growth parameters is fundamental in achondroplasia. Height, weight, and head circumference should be measured every 3–6 months and plotted on achondroplasia-specific growth charts, as standard population charts can underestimate AGV and misidentify complications such as hydrocephalus or obesity (1, 14, 18). Consistent use of validated reference data helps clinicians track deviations that may signal spinal or neurologic issues requiring early intervention (15, 23).

Measurements should be performed consistently by the same trained clinician or nurse wherever possible, using standardized techniques to ensure reliability. Inaccurate or inconsistent anthropometry can lead to misinterpretation of growth trends. It is recommended that anthropometrists develop and use an institutional standard operating procedure to ensure uniform technique, staff training, and equipment calibration. Furthermore, if measurement adaptations are needed due to body disproportions, joint contractures, bowing, or spinal deformities, these must be documented to ensure longitudinal consistency (24).

#### 4.3.2 Regular screening

Regular, proactive surveillance is essential given the multi-system comorbidity burden in achondroplasia. In infancy and early childhood, clinicians should closely monitor for craniocervical junction compromise (via neurologic examination, growth/head-circumference trajectory, and targeted neuroimaging when indicated) and screen for sleep-disordered breathing, with symptom review and polysomnography when clinically warranted; both problems carry meaningful morbidity risk if missed (22). ENT/audiology follow-up is recommended to detect recurrent otitis media and conductive hearing loss that can affect speech-language development (14). Lifelong orthopedic surveillance (thoracolumbar kyphosis, progressive spinal stenosis, genu varum) should be integrated into routine visits, alongside neurological assessment for signs of cord/root compression (1, 15). Finally, weight management and nutrition counseling should be embedded into care using diagnosis-appropriate anthropometry and BMI interpretation, as excess adiposity and sleep apnea are linked to adverse cardiometabolic and functional outcomes in achondroplasia (24).

### 4.4 Pharmacological treatment

#### 4.4.1 Treatment with vosoritide

Vosoritide, a CNP analog that counteracts overactive FGFR3 signaling, should be considered in eligible children from birth or 4 months of age, consistent with local regulatory labels and the latest treatment guidelines. In a landmark clinical phase III extension study, vosoritide led to a height gain of 5.75 cm more than untreated children and significantly improved upper-to-lower body segment ratio at 3 years (11). Real-world cohorts reinforce these trial findings: the French early-access program (n=57) reported a mean AGV of 6.0 cm among patients aged 5 years or older, with only mild adverse events (mostly injection-site reactions) and no discontinuations; a German single-center case series (n=34) reported a significant increase in height z-score and 6-minute walk distance improvement at 12 months; and a Portuguese cohort (n=27) of children with achondroplasia aged 2 to 14 years reported a mean AGV of 5.87 cm per year and no serious adverse events or discontinuations (10, 25, 26).

While the available evidence supports the efficacy and safety of vosoritide in improving growth velocity and body proportionality, several important uncertainties remain. Long-term data on final adult height, functional outcomes, QoL, durability of treatment benefit, and cost-effectiveness are still emerging. In addition, daily subcutaneous administration may represent a treatment burden for some children and families, potentially affecting long-term adherence. Continued post-marketing surveillance and real-world studies are therefore needed to further characterize long-term safety and clinical outcomes. Decisions regarding treatment initiation should be individualized and guided by shared decision-making, taking into account available evidence, local regulatory approvals, family preferences, treatment burden, and healthcare system considerations.

Initiation of vosoritide therapy should follow a structured and safety-focused approach. Caregivers should receive comprehensive injection training delivered by a specialist nurse, including education on correct administration technique and systematic injection-site rotation. It is recommended that the first dose be administered under medical supervision, with an observation period of approximately 1 hour to monitor potential adverse reactions, particularly transient hypotension (27). Families should also be advised to ensure adequate hydration prior to injection, as this has been shown to mitigate the risk of vasodilatory effects associated with CNP analogs. These measures are essential to promote safe initiation, caregiver confidence, and long-term adherence (27). **Table 7** lists a summary of side effects reported with vosoritide in children with achondroplasia. The primary safety data are derived from clinical trials and real-world studies in pediatric populations (11, 26, 28–31).

**Table 7:** Summary table of side effects reported with vosoritide in children with achondroplasia.

Side effect	Description	Source types
Injection-site reactions (11, 28)	Transient swelling and/or erythema at the site of subcutaneous injection; this is one of the most commonly reported adverse events in trials and real-world use.	Clinical trial; clinical trial extension phase
Injection-site pain / discomfort (11, 28)	Pain at the injection site sometimes requiring local measures; part of the spectrum of injection-site reactions.	Clinical trial; clinical trial extension phase
Transient hypotension / blood pressure changes (11, 29)	Brief decreases in blood pressure after dosing have been observed (typically mild and self-limited); associated symptoms may include dizziness and nausea.	Clinical trial; published review
Pyrexia (11, 28, 30)	Fever reported among adverse events in clinical trials and systematic safety reviews of vosoritide; typically mild and transient.	Clinical trial; clinical trial extension phase; systematic review
Hypertrichosis (26, 31)	A highly uncommon side effect that has recently been reported; excessive hair growth was not initially reported in pivotal trials but recognized in post-marketing and observational data.	Single-center observational study; case report

Published consensus guidelines and reviews delineate eligibility (open epiphyses) and administration/dosing requirements, and reinforce the importance of family training/adherence and multidisciplinary monitoring. Collectively, these data support offering vosoritide from birth or 4 months of age (as per local regulatory approvals and available clinical evidence) in appropriate candidates to enhance growth and body proportionality with acceptable tolerability, while emphasizing the need for regular monitoring of treatment response using standardized anthropometric measurements (12, 19, 27, 29). We recommend conducting monitoring assessments every 3 months in children aged 0–2 years, every 4–6 months in children aged 3–5 years, and every 6 months in children older than 5 years to guide long-term management and optimize outcomes.

#### 4.4.2 Promoting equitable access to therapy

In the Middle East, access to rare disease therapies such as vosoritide is often influenced by structural and socioeconomic disparities within national healthcare systems. While nationals in several countries in the region benefit from government-funded healthcare and reimbursement, expatriates, who constitute a large proportion of the regional population, typically rely on private insurance plans that may not cover high-cost orphan drugs. In other countries in the region, including those where vosoritide is not yet routinely provided through public healthcare systems, access may be limited to families who can afford the financial, administrative, and time burdens associated with legally obtaining the therapy, creating inequities even among citizens. These disparities risk creating significant treatment gaps in children with achondroplasia. Clinicians therefore have an important role in advocating for equitable access and reimbursement mechanisms, ensuring that eligibility for vosoritide is guided by clinical need rather than nationality, insurance status, or ability to self-fund treatment. Promoting collaboration between public health authorities, insurers, and patient advocacy groups can help establish sustainable funding pathways that uphold fairness and optimize outcomes for all children with achondroplasia in the region.

#### 4.4.3 Treatment cessation and shared decision-making

Clear guidance on treatment cessation is important to ensure appropriate use of vosoritide and continuity of care beyond active therapy. As linear growth potential diminishes with advancing skeletal maturity, continuation of treatment is unlikely to confer additional benefit once AGV falls below clinically meaningful thresholds or when epiphyseal closure is confirmed radiographically. International guidance and expert opinion support discontinuation under these circumstances, provided adherence and dosing have been adequate (19, 27). Importantly, cessation of pharmacologic therapy does not mark the end of clinical follow-up. Individuals with achondroplasia remain at risk of progressive spinal, orthopedic, and neurological complications into adolescence and adulthood. Ongoing surveillance, coupled with a structured transition to adult services, is therefore essential to maintain long-term health, functional independence, and QoL (12, 27, 29).

Pharmacologic decisions in achondroplasia, including vosoritide initiation and cessation, should be guided by shared decision-making between clinicians and families, ensuring that treatment choices reflect medical evidence as well as family expectations and contextual realities. International guidelines emphasize that initiating therapy requires detailed discussions about benefits, limitations, monitoring requirements, and long-term uncertainties (1, 19, 27). Families should be empowered with balanced, comprehensible, and culturally sensitive information to make informed decisions aligned with their child’s best interests. In the Middle East, this process must also consider financial constraints, insurance coverage, and healthcare system variability, which can influence access to therapy and adherence. Embedding structured counseling and multidisciplinary input within the decision-making process fosters trust, supports adherence, and promotes family-centered, comprehensive care.

### 4.5 Long-term management and transition to adult care

#### 4.5.1 Follow-up and use of QoL tools

Long-term follow-up in achondroplasia should be structured, risk-based, and multidisciplinary, with periodic assessments tailored to age and clinical status. International consensus guidelines recommend surveillance across key organ systems, including neurological, respiratory, ENT, orthopedic, dental, and psychosocial domains, to ensure early detection and management of complications such as foramen magnum stenosis, sleep apnea, otitis media, spinal stenosis, and obesity (1, 14, 15). Clear follow-up protocols would enhance care continuity, particularly when coordinated by a dedicated clinician or multidisciplinary team.

Furthermore, QoL is an essential component of longitudinal care in achondroplasia, given the well-documented impacts on physical functioning, daily activities, and psychosocial well-being. Evidence consistently shows that children and adults with achondroplasia experience higher rates of physical limitations, social difficulties, and emotional challenges compared to average-stature peers (9). Routine use of validated QoL instruments, such as the PedsQL, QoLISSY, and newer condition-specific tools, allows clinicians to identify domains requiring support and monitor change over time. However, for meaningful implementation across the Middle East region, there is a need to develop, translate, and validate culturally appropriate and linguistically adapted QoL instruments that reflect regional social norms, family structures, and health perceptions. Broader literature on short-stature disorders confirms that multi-dimensional QoL assessments capture outcomes not reflected in clinical measures alone and are critical for tailoring interventions to individual needs (32). The availability of regionally relevant QoL tools would further enhance the interpretability and clinical utility of longitudinal assessments, supporting a more holistic care approach that addresses emotional resilience and social integration alongside medical care.

#### 4.5.2 Establishing support groups

Support groups can support the long-term care of individuals with achondroplasia by fostering information sharing, emotional support, and social connection among families who often face similar medical, psychosocial, and practical challenges. International guidelines highlight the value of integrating patient and family advocacy organizations into routine care pathways (1, 6, 14, 15). In the Middle East, families frequently rely on informal networks due to limited availability of structured psychosocial support, underscoring the need to establish and strengthen family-led peer support groups that are culturally sensitive and accessible. Empowering these groups, through clinician collaboration, educational resources, and inclusion in care planning, can enhance community resilience and support shared decision-making.

#### 4.5.3 Dedicated transition clinics

A structured transition from pediatric to adult care is essential in achondroplasia, as individuals continue to face orthopedic, neurological, respiratory, and psychosocial challenges throughout adulthood. International guidelines recommend formal transition pathways to maintain continuity and address evolving needs (1, 14, 15). However, in the Middle East, the transition phase remains a significant unmet need due to fragmented care systems, limited adult multidisciplinary expertise, and logistical barriers that can result in loss to follow-up and gaps in long-term monitoring. Establishing coordinated transition programs, supported by clear referral pathways, structured counseling, and collaboration between pediatric endocrinologists, adult specialists, and allied health professionals, can help young adults with achondroplasia navigate this critical period with greater safety and autonomy. **Figure 1** illustrates a structured, multidisciplinary pathway from initial diagnosis through early risk stratification, shared decision-making, pharmacologic treatment with vosoritide (where appropriate), long-term surveillance, and transition to adult care. The pathway emphasizes standardized assessments, safety monitoring, psychosocial support, and continuous care across the lifespan, aligned with international guidance and regional considerations in the Middle East.

**Figure 1:**
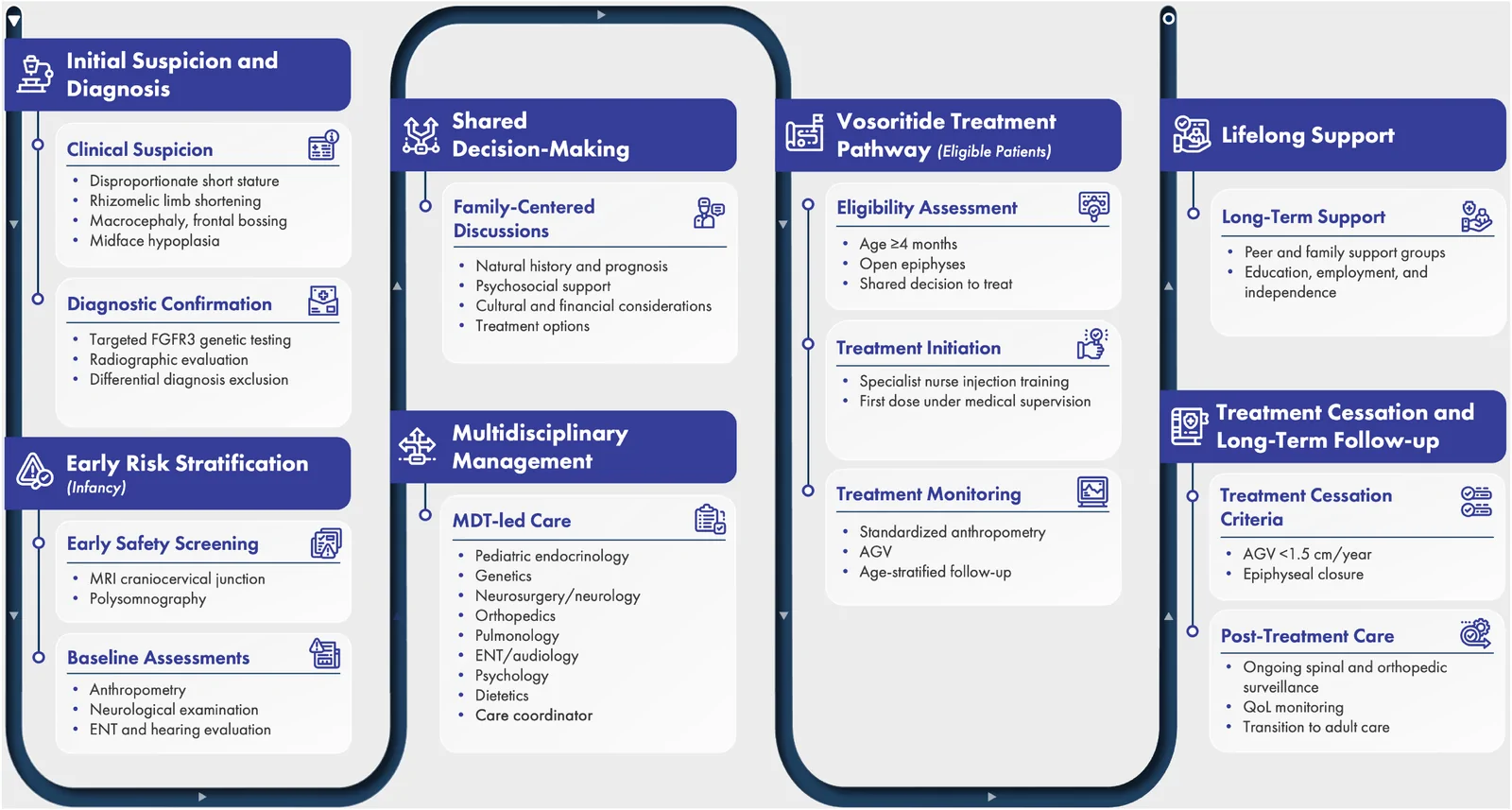
Consensus-based pathway of care for individuals with achondroplasia across the lifespan. AGV, annualized growth velocity; ENT, ear, nose, and throat; QoL, quality of life.

Beyond transition itself, adult care should address the ongoing medical, functional, and psychosocial challenges associated with achondroplasia. Common adult complications include spinal stenosis, chronic pain, obesity, sleep-disordered breathing, reduced mobility, and mental health concerns, all of which may impact QoL and independence (33). Adult-focused services should also provide counseling on employment, social participation, reproductive health, family planning, and long-term functional support where required. Establishing collaborative care pathways between adult physicians, orthopedic specialists, neurologists, pain specialists, rehabilitation teams, and mental health professionals may help ensure continuity of care and optimize long-term outcomes for adults living with achondroplasia.

### 4.6 Real-world data and registries

#### 4.6.1 Facilitating data collection and reporting

Establishing a regional achondroplasia data registry, or contributing to existing international registries, is essential to strengthen evidence generation and enable standardized data collection. This would also allow comprehensive evaluation of the long-term safety and efficacy of emerging therapies such as vosoritide. In the Middle East, where epidemiological data remain limited and healthcare systems vary widely, a unified registry would enhance research capacity, inform clinical guidelines, and facilitate collaboration across countries in the region.

#### 4.6.2 Conducting observational studies

Retrospective, observational studies using pre-existing clinical data can be a practical approach to generating real-world evidence on achondroplasia in the Middle East. Given the variability in healthcare access and follow-up pathways across the region, leveraging existing medical records can provide valuable insights into local disease patterns, comorbidity burden, treatment uptake, and long-term outcomes. Such studies require minimal additional infrastructure and can be implemented across multiple centers, helping to build a foundation of regional data that can inform clinical guidelines, support policy decisions, and complement prospective research and registry initiatives.

### 4.7 Strengths and limitations of the consensus

This consensus represents the first region-specific effort to develop practical recommendations for the diagnosis and management of achondroplasia in the Middle East and Egypt, addressing an important unmet need in a rare disease area where regional guidance is limited. The recommendations were developed using a structured modified Delphi methodology informed by a targeted literature review, international guidance documents, and expert clinical experience. The consensus addresses the full continuum of achondroplasia care, including diagnosis, surveillance, multidisciplinary management, psychosocial support, pharmacological therapy, transition to adult services, and real-world evidence generation, while also considering challenges relevant to healthcare systems in the region.

However, several limitations should also be acknowledged. The expert panel consisted primarily of pediatric endocrinologists and clinical geneticists, and therefore this work should be considered an endocrinology/genetics-led regional consensus rather than a comprehensive multidisciplinary clinical guideline. Although the recommendations emphasize the importance of multidisciplinary care, direct participation from other specialties, including orthopedic surgery, neurosurgery, pulmonology, otolaryngology, rehabilitation medicine, psychology, and adult medicine, was not included in the Delphi process. In addition, the panel represented several countries within the Middle East region, but not all countries were represented, and healthcare systems, resource availability, reimbursement mechanisms, and access to specialist services vary considerably across the region. As such, some recommendations may require adaptation to local healthcare contexts. Furthermore, patients, caregivers, and patient advocacy organizations were not directly involved in the development or voting process. Future consensus initiatives would benefit from broader multidisciplinary, geographic, and patient/family representation to further strengthen the relevance and applicability of the recommendations.

## 5 Conclusion

This regional consensus, endorsed by the Arab Society for Pediatric Endocrinology and Diabetes (ASPED) and the MENA Medical Genetics Association, provides a comprehensive, evidence-informed framework for the holistic management of achondroplasia, addressing diagnosis, surveillance, multidisciplinary care, psychosocial support, and emerging therapeutic options. By integrating global best practices with the unique healthcare realities of the Middle East, these statements aim to standardize care, reduce variability, and improve outcomes. The statements presented reinforce the importance of early detection, coordinated multidisciplinary follow-up, shared decision-making, and equitable access to treatments such as vosoritide. They also highlight critical system-level needs, including structured transition pathways, strengthened support networks, and enhanced real-world evidence generation through registries and observational studies. Collectively, this consensus provides a practical regional framework to support more consistent and patient-centered achondroplasia care across the Middle East.

## Data Availability

The original contributions presented in the study are included in the article/supplementary material. Further inquiries can be directed to the corresponding author.
